# Automated population‐based planning for whole brain radiation therapy

**DOI:** 10.1120/jacmp.v16i5.5258

**Published:** 2015-09-08

**Authors:** Eduard Schreibmann, Tim Fox, Walter Curran, Hui‐Kuo Shu, Ian Crocker

**Affiliations:** ^1^ Department of Radiation Oncology Emory University School of Medicine Atlanta GA USA

**Keywords:** knowledge‐based planning, automation, whole brain planning

## Abstract

Treatment planning for whole‐brain radiation treatment is technically a simple process, but in practice it takes valuable clinical time of repetitive and tedious tasks. This report presents a method that automatically segments the relevant target and normal tissues, and creates a treatment plan in only a few minutes after patient simulation. Segmentation of target and critical structures is performed automatically through morphological operations on the soft tissue and was validated by comparing with manual clinical segmentation using the Dice coefficient and Hausdorff distance. The treatment plan is generated by searching a database of previous cases for patients with similar anatomy. In this search, each database case is ranked in terms of similarity using a customized metric designed for sensitivity by including only geometrical changes that affect the dose distribution. The database case with the best match is automatically modified to replace relevant patient info and isocenter position while maintaining original beam and MLC settings. Fifteen patients with marginally acceptable treatment plans were used to validate the method. In each of these cases the anatomy was accurately segmented, but the beams and MLC settings led to a suboptimal treatment plan by either underdosing the brain or excessively irradiating critical normal tissues. For each case, the anatomy was automatically segmented with the proposed method, and the automated and manual segmentations were then compared. The mean Dice coefficient was 0.97, with a standard deviation of 0.008 for the brain, 0.85±0.009 for the eyes, and 0.67±0.11 for the lens. The mean Euclidian distance was 0.13±0.13 mm for the brain, 0.27±0.31 for the eye, and 2.34±7.23 for the lens. Each case was then subsequently matched against a database of 70 validated treatment plans and the best matching plan (termed autoplanned), was compared retrospectively with the clinical plans in terms of brain coverage and maximum doses to critical structures. Maximum doses were reduced by a maximum of 8.37 Gy for the left eye (mean 2.08), 11.67 for the right eye (1.90) and, respectively, 25.44 (5.59) for the left lens and 24.40 (4.85) for the right lens. Time to generate the autoplan, including the segmentation, was 3−4 min. Automated database‐ based matching is an alternative to classical treatment planning that improves quality while providing a cost‐effective solution to planning through modifying previous validated plans to match a current patient's anatomy.

PACS number: 87.55.D, 87.55.tg, 87.57.nm

## I. INTRODUCTION

Information available in the form of previous clinical cases used to treat similar anatomy is increasingly used in radiotherapy to judge the quality of new plans or to suggest constraints for the inverse planning process. Generally termed knowledge‐based treatment planning, this information becomes largely available with the informatization of radiotherapy practice in the comprehensive database systems that are the basis for modern software in the treatment planning process. Our effort is encouraged by recent reports showing that integrating *a priori* information into the treatment planning process is efficient in IMRT. Indeed, incorporation of prior knowledge in the treatment planning process^1^, ^2^ is an effective method to reduce the lengthy times^(3)^ associated with selecting optimal beam configurations^4^, ^5^ and dose‐volume constraints.^(6)^ Wu and colleagues^7^, ^8^ investigated the use of overlaps between critical structures and the planning volume for predicting achievable DVH. Similarly, Zhu et al.^(9)^ used the database of previous plans to derive a set of attainable DVHs that are grouped together by anatomical similarity and, with principal component analysis (PCA) used on the DVH, quantified their noticeable features as a dose prediction tool for prostate cases treated with IMRT. Geometry‐based approaches that consider individual patient topology have been also used to create maps that describe a specific direction to access a tumor without intersecting critical organs.^10^, ^11^ When incorporated into the treatment planning process,^3^, ^4^, ^12^, ^13^ this *a priori* information improves planning times as the algorithm employs favorable beam settings from the geometrical location of critical structures relative to the PTV.

Whole‐brain radiotherapy planning aims to deliver the prescribed dose of radiation uniformly to the brain while minimizing the dose spill to the nearby eyes and lenses. It is typically employed for patients who have multiple metastases scattered throughout the brain. Technically, this is usually achieved by a placing two opposing open‐field lateral beams and shaping the beam aperture with the multileaf collimator (MLC) in the brain–eye interface to block dose delivery to these critical organs. Leakage from brain fields to eyes and lenses cannot be intuitively accounted for; therefore, finding the optimal MLC positions depends on patient geometry with a few rounds of trial and error testing being needed to find their optimal positions. Other manual steps that consume valuable clinical time are segmenting the brain and critical structures, as well as various software steps needed to define the plan, insert the beams, and configure the MLC settings.

We hypothesized that many of the manual tasks involved in treatment planning for this condition could be automated to create a clinical workflow where a plan is ready for review in a few minutes after a simulation CT of the patient was acquired. The approach presented in the following focuses on whole‐brain radiation therapy, a treatment where the position and shape of target and critical structures varies little from patient to patient and facilitates our endeavor. Automating the segmentation is facilitated by the fact that for this disease site organs are relatively identifiable from surrounding anatomy and have minor variance across patients. Similarly, for the planning process, the target volume can be defined on the CT scan alone and delivery involves 3D conformal technique that is technically less demanding when compared to other modern techniques. Indeed, segmentation of brain and eyes from CT datasets is clinically available in commercial software and is of potential use to automate the process. However, setting of the MLC position is an intuitive task that requires human knowledge and cannot be easily quantified in mathematical terms as basis for an automated procedure.

In the framework of gaining knowledge from clinical databases, we sought to replicate a dosimetrist's or physician's experience in setting MLC positions for brain planning by searching the information within the database to query and retrieve previous cases of similar anatomy. In the proposed database mining approach, automatically generated contours of the patient's segmentation are used as an input and then compared to previously treated patients to find a patient with similar anatomy. The associated clinical plan is then reused for the new patient by changing DICOM information within the old plan to match the new patient's name and ID, while keeping the original treatment settings. Upon reformatting the DICOM tags, a plan and associated segmentation are imported in the treatment planning system for review after the prescription dose is specified and the dose is recomputed. Although the anatomy is not identical, it is still similar enough on patients matched by this procedure such that minor anatomical differences do not produce clinically significant dosimetric consequences. If true, dose recomputation and plan review are the only tasks that require human intervention in the whole planning process.

The novelty and critical aspect of our endeavor is the metric used to find similar case in the database. The metric is a single scalar value that quantifies the degree of matching between two cases. The technical difficulty arises as the metric must capture and describe relevant features fast enough to enable comparison to hundreds of cases in a few minutes. To better detect the geometric variations that contribute to target underdose, we developed a customized measure comparing position and shape of target and critical structure only near the MLC positions and perpendicular to the beam directions. In the following, we present the technical aspects of the metric construction and search procedure, while illustrating its application in routine clinical operations.

## II. MATERIALS AND METHODS

**Figure 1:**
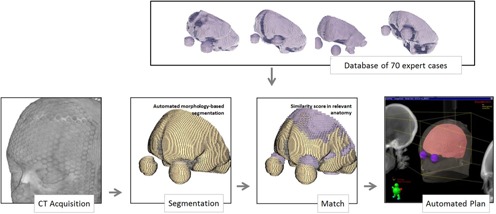
Scheme of the proposed autoplanning procedure. Input is only the CT dataset. An autosegmentation procedure detects the brain and critical structures. This segmentation is matched against a database of high‐quality plans to find the closest anatomical match. The plan associated with the closest match is assigned to the new patient and send to the treatment planning system for review.

A scheme of the overall procedure is presented in Fig. 1, detailing the input and algorithms proposed for whole‐brain planning. Inputs are the CT scan of a new patient and an expert database of high‐quality plans of patients treated in our clinical practice. Upon reviewing past treatment plans, data were saved in a plain directory and subsequently used as search database in this study. When a new patient is simulated, the CT dataset is send to an in‐house software module that automatically segments brain, eyes, and lenses and then matches this segmentation to the database to find the plan with the closest geometric resemblance. The beam settings and MLC positions for the best match are retrieved, reformatted, and saved as a new plan that is imported in the treatment planning system; the dosimetrist can review, modify, and recompute dose in the new plan. The technical details of the segmentation procedure and database matching are given in the Materials & Methods sections C and D, respectively.

### A. Test cases

In the database of patients treated at our institution we encountered 15 cases in which the anatomy was accurately segmented, but the beams and MLC settings employed led to a suboptimal treatment plan by either underdosing the brain or by excessive irradiation of critical normal tissues. We would like to point out that only plans considered suboptimal upon review were used as test cases in the following. The overall intent for the comparison, shown in the Results section, was to verify that the algorithm can detect and modify errors in these plans as a proof of principle that automated planning can surpass possible human errors due to lack of experience. If the automated tool is adopted in the clinic, the gain obtained in plan quality, as shown in the Results section, would be diminished if experienced dosimetrists plan the test cases.

### B. Database creation

A set of 70 cases were reviewed and selected by one of the authors (IC), a physician with 31 yrs of experience in brain treatments, and saved as DICOM files in a plain hard drive directory. As the matching is performed on the structure sets only and does not include any image features, the CT datasets and any other information are discarded to significantly reduce database size and accelerate the search procedure. The plans are saved, along with the segmentation, as they are used as templates to create new plans after reformatting some information within to assign them to new patients.

### C. Segmentation

**Figure 2:**
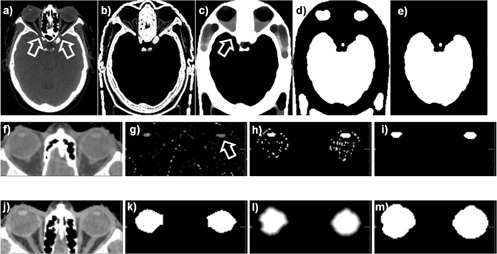
Illustration of the grayscale processing and morphological filters used to detect the brain (upper row), lens (middle row), and eye (lower row). Technical details are given in the Materials & Methods section C.

Segmentation is achieved by automatically applying a combination of imaging filters that detect and extract the soft tissue voxels followed by a combination of morphologic operators that characterize the soft tissue shapes to select the blobs representing organs of interest based on characteristics such as volume or roundness. Technical details and the operators used are detailed in Fig. 2, where the upper row shows the steps necessary to extract the brain, eye, and lens contours. While the brain can be characterized as the organ with the largest volume within the CT scan, the eyes are detected as the roundest soft tissue shapes in the CT datasets, and the lenses are detected as the largest patches of higher HU units inside the brain. The method uses standard filters to isolate soft tissue voxels and process them to find the largest or roundest patch. A set of intermediary image filters are used as intermediary steps to avoid noise or preprocess the data. For example, the brain is the largest organ but is also connected to other soft tissue such as the extraocular muscles, as indicated by arrows in the figure. These connections are eliminated by a binary erosion filter that erases connections that are smaller than a user defined size, before a quantification filter identifies the brain as the largest patch of soft tissue. Overall, the procedure starts with a classical thresholding algorithm that extracts voxels of HU units in the range of −200 to 200, corresponding to soft tissue. The procedure follows then by a set of filters that were configured experimentally to extract the organs of interest in this project, these specific filters being a Sobel edges^(14)^ to detect bone in the image (Fig. 2(b)), a grayscale closing filter to connect and close the holes at the arrows (Fig. 2(c)) to create a mask (Fig. 2(d)) that is subsequently applied on the original image to isolate soft tissue paths (Fig. 2(e)). The largest of these patches by volume is retained as the brain segmentation (Fig. 2(f)). Similarly, to segment the lens (second row in Fig. 2) the roundest two regions in the soft tissue mask (Fig. 2(e)) are used to extract a volume of interest around the eyes (Fig. 2(f)). An H‐Convex algorithm^(15)^ isolates the regions in the CT dataset that have a HU value above a threshold from its neighboring voxels (Fig. 2(g)), with subsequent processing isolating the largest path (Fig. 2(h)) and applying a binary closing filter to smooth it (Fig. 2(i)). The eye segmentation erases the lens intensities to background value in the CT dataset (Fig. 2(j)), erodes the largest roundest region in the soft tissue mask (Fig. 2(d)) to eliminate connectivity to other soft tissue (Fig. 2(k)), applies a Laplacian level set filter to adapt the mask to small details in the CT dataset (Fig. 2(l)), and finally applies a binary closing to create a smooth final segmentation (Fig. 2(m)).

Any segmentation algorithm can be used for this step if it provides a clinically accurate segmentation of the critical structures, alternative approaches being implemented in various commercial or open‐source solutions. The above process does not require any intervention by the user, the only input to the segmentation procedure being the patient's CT. This straightforward computational setup constitutes the main reason it was selected over alternative algorithms that can segment brain structures, such as atlas‐based segmentation approaches.^16^, ^17^

Output is a segmentation of the structures of interest in DICOM format that was compared with the clinical segmentation using the classical Dice and Hausdorff measures. The Dice measures the degree of overlap between the automated and clinical segmentation ranging in value from 0 when the structures do not overlap at all to 1 for a perfect match. This measure is used to assess the spatial correspondence of the segmentations and empirically denotes a good segmentation if it fails above a value of 0.8, with higher numbers denoting a better match. The Dice measures the coincidence in terms of shapes intersection and union and, for smaller structures, may overestimate the error. The Hausdorff distance measures the largest distance between two surfaces, but not the agreement in overall shape characteristics.

### D. Database matching

**Figure 3:**
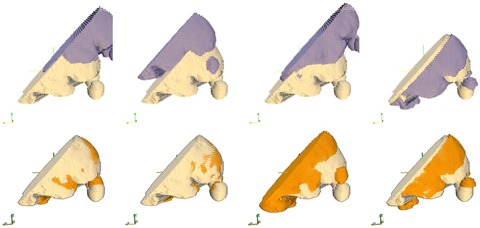
Examples of match between the patient and database segmentations. Each column shows matching of the patient contours (light yellow) to a different database case before (upper row) and after (lower row) an ICP algorithm that corrects for isocenter positioning. Remaining anatomical discrepancies visible in the lower row are quantified to select the closest database plan. For the database cases shown, the second column provided the best match, while the third column had the worst. Note that matching is only on relevant anatomy close to the MLC and discards posterior and superior brain anatomy that is irradiated by an open field in this technique.

Segmentation obtained in the previous process is used as input to match the patient to a database case. In the standard technique, much of the brain is irradiated by an open field and thus the anatomical match in this region is less relevant. In our study, we discarded the posterior and superior brain contours by cropping the brain segmentation with a plane oriented from the nape toward the forehead, as shown in Fig. 3, to focus our calculations on the brain–eye region where the eyes are protected by the MLC creating a high‐dose gradient where shapes have to match precisely.

Segmentations to be compared are not spatially aligned as the patients may be set up at simulation in different positions on the CT couch. An iterative closest point (ICP)^18^, ^19^ algorithm was employed to align the patient and database segmentations to minimize the distance between the points defining the two surfaces. The ICP algorithms starts by aligning the center of mass of the segmentations, then iteratively modifies a transform that considers translations on all three axes to minimize the point‐by‐point distance between the datasets to be aligned. The procedure is illustrated in Fig. 3 where the upper row shows the surfaces before alignment, while the lower row shows the same surfaces after being aligned by the procedure. The ICP algorithm eliminates the initial bulk displacement by spatially aligning the surfaces to leave only intrapatient geometrical variations that can now be quantified to characterize the similarity between the patient and database cases.

This similarity between the new case and a database case is quantified by a number, called in the following score. We have chosen to mathematically characterize the similarity as the squared distance from the patient to the database segmentation, computed and averaged on every point defining the brain and eyes. Because the planning setup involves simple lateral opposed beams with limited separation, geometrical differences along the beam direction were not expected to contribute significantly to the final dose deposition. In contrast, geometric changes across the beam direction in the region shaped with the MLC are critical and could result in underdosing of the target or overdosing of critical structures. To make the metric sensitive to relevant geometric changes only, the anterior–posterior and superior–inferior components of the distance that are oriented across the beam direction are considered in the metric computation, while left–right variations are discarded. Overall, the score was defined as:

(1) $$score=sqrt(\sum_{i=1}^{N}({\left(y_{i}-closest(y_{i})\right)}^{2}+(z_{i}-closest{\left(z_{i}\right)}^{2}$$

where *score* represents the score value used to judge the similarity of the database to new case segmentations; the sum is over all points defining the new case segmentation, taking the squared difference of the x and z components of these points to the closest points in the databases case; and the *closest*($y_{i}$) represents the closest point in the database segmentation to the ith point in the new cases segmentation. The sum considers only the y and z components that are perpendicular to the field, with the x component of this distance being ignored, as detailed above.

In our initial tests we also noted that large local discrepancies can have a significant effect on the dose, while constant small discrepancies do not significantly affect it. In a hypothetical example, a match that has low discrepancies of less than 1 mm everywhere except a local region with a high discrepancy of 7 mm will produce significant dose disturbance at the discrepancy leading to a clinically unacceptable plan. For comparison, a constant discrepancy of 2 mm everywhere is likely to produce a clinical acceptable plan, as the dose distribution's shape remains unchanged. When comparing these two hypothetical solutions with a standard metric computing the mean distance, the first scenario gives a smaller mean value, indicating erroneously a better match. To overcome this behavior, we use the squared distance in the metric computation to penalize larger anatomical discrepancies over smaller ones.

## III. RESULTS

### A. Segmentation

**Figure 4:**
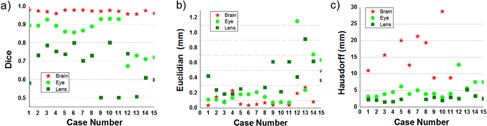
Comparison of automated and manual segmentation through Dice coefficients (a), Euclidian distance (b), and Hausdorff distance (c). An ideal Dice coefficient is 1, the higher the better, while the ideal Hausdorff distance is 0, the lower the better.

**Figure 5:**
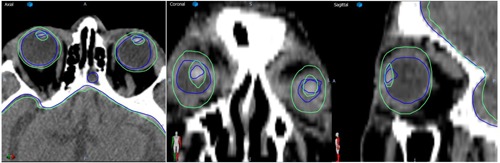
Example of automated (green) and manual (blue) contours for a case that performed worse (#13) according to the criteria in Fig. 4.

Differences between the manual and automated segmentation of the test cases are quantified in Fig. 4 which plots the Dice coefficient and the Euclidian and Hausdorff distance for brain, eye, and lens in all 15 cases. The Dice coefficient for the brain ranged between 0.956 and 0.979, with a mean at 0.970 and a standard deviation (SD) of only 0.008. For the eyes, we have plotted the worst value in either left or right eye, the values ranging between 0.673 and 0.930, with a mean at 0.846 and an SD of 0.090. The lens being a smaller organ is the most sensitive to disagreements between the manual and automated segmentation; however, the values remained clinically useful with values ranging from 0.501 to 0.800 with a mean of 0.672 and an SD of 0.501. The Dice index tends to overestimate the error for smaller structures as their volume is small and thus smaller discrepancies create lower values for this metric. However, these small discrepancies can often be overlooked or easily corrected in clinical practice. In Fig. 5 we show the resulting segmentation for the Case #13 that is one of the worst‐performing cases, according to the Dice coefficients for eye and lens.

The mean Euclidian and Hausdorff distances were also measured. The Hausdorff distances are another estimator of the time needed to correct automated contours, measuring the maximum discrepancy between each point in the automated segmentation to the closest point in the clinical one. The lower this distance, the better contours agree. When comparing the clinical and automated segmentations, as shown in Figs. 4(b) and (c), the mean Euclidian distance ranged between (0.03, 0.49) for the brain, (0.07, 1.15) for the eye, and (0.18, 27.43) for the lens. Overall the Euclidian distances are below 0.5 mm for all structures in all cases, with occasional mean discrepancies of less than 1 mm recorded for eye and lens in a few cases. The Hausdorff distances are shown in Fig. 4(c), ranging between (8.75, 116.16) for the brain, (2.92, 12.70) for the eye, and (1.51, 60.56) for the lens. Overall these discrepancies are below 10 mm for eye and lenses, with larger value for the brain. Mean and SDs were 0.1252608 and 0.121 for the brain, 0.27 and 0.31 for the eye and, for the lens, 2.34 with an SD of 7.23. On the Hausdorff distance, the mean and SD were 33.19 and 28.11 for the brain, and 5.18 and 2.54 for the eye, while, for the lens, the values were 6.72 and 15.53.

**Figure 6:**
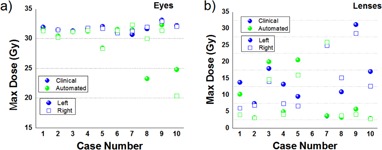
Comparison of maximum doses for critical structures. The graphs show the maximum doses in the clinical (blue) and automated plans (green) for eyes (a) and lenses (b). The dose to the eye is lower or comparable in the autoplan solution, while for the lens it decreased in 6 out of 10 cases. Spheres represent the left eye or lens, squares represent their right counterparts.

A typical segmentation that performed worse is presented in Fig. 5 which shows the differences between the automated (green) and clinical (blue) segmentations as overlaid on the CT dataset. For this particular case, the clinical eye segmentation is somehow interior to the eye, while the automated one oversegments in the superior–inferior direction. Similarly the lens segmentation is undersized in the clinical segmentation and overly generous in the automated one. For most cases, the agreement was better as this figure shows one of the worst results. The Dice coefficient does not necessary mean a perfect match in the segmentation, as seen for the brain in the same Fig. 5 where differences of a few mm between the automated and manual segmentations are visible despite the good value of the Dice coefficient for this case. This can be explained by the large volume of the brain that washes out small errors in the segmentation. For a complete analysis, we reported also the Euclidian and Hausdorff distance in Fig. 4 that specifically catches the maximum segmentation discrepancies independent of the organ volume. As this is one of the cases used in the evaluation on Fig. 6, for this particular case this discrepancy did not create big dosimetric differences, but for other situations such a discrepancy may lead to suboptimal planning on other cases. Although the planning is automated, a physician still has to review and approve the contours and plan. There will be instances where the automated planning will be clinically inacceptable and manual editing will be required. Clinical day‐to‐day implementation and testing of this segmentation approach will validate or invalidate it from a practical point of view.

### B. Database matching

**Figure 7:**
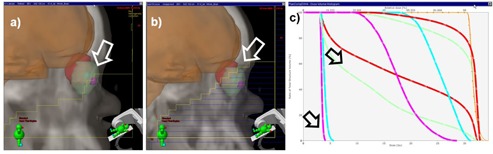
Comparison of the original clinical (a) and the autogenerated (b) plans. The white arrows show differences in MLC positions (shown as yellow and blue lines) with the automated plan blocking more of the upper eye region. The DVHs comparison in (c) shows that for the same PTV coverage, the dose received by eyes and lens is lower in all aspects for the autoplan solution (marked with arrows) when compared to the clinical plan.

A comparison of a marginally acceptable clinical plan and the automated plan is shown in Fig. 7, where the MLC positions are shown as blue‐yellow lines superimposed on the eye (green and red spheres) and brain (orange surface). One item of note is that, on the clinical plan (left), the MLCs too generously encompassed the brain, resulting in a larger volume of the eye and lenses receiving an unnecessarily high dose. The MLC solution, proposed by the database match, positions the MLC segments closer to the brain, thus protecting more of the eye and lenses. The DVHs for the clinical and autoplanned solutions are shown in Fig. 7(c), with the DVHs being almost identical for the brain but with significant sparing obtained for both eyes and lenses. Comparing the two, the autoplaned solution irradiated only 46.5% and 20.8% of the left and right eyes at 20 Gy (red and green lines in the DVH), while the clinical plan irradiated 96.3% and 90.3% at the same isodose level. Likewise, the maximum dose of 31.71 and 28.5 Gy to the left and right lenses in the clinical plan was reduced almost six times to 5.7 and 4.1 Gy in the automated solution (violet and blue lines in the DVH).

A summary of the dose reduction for all organs in the clinical cases judged as suboptimal by their maximum dose to critical structures is shown in Fig. 6. For all cases presented, the dose sparing provided by the autoplan solution in the eyes was better or equal to the clinical plan, with marked reductions observed in some cases. For example, maximum dose (Fig. 6(a)) for Case #10 decreased from 32.2 to 24.8 in the left eye and 32.1 to 20.3 Gy in the right eye. Similarly, the maximum dose to the lens (Fig 6(b)) decreased from 17.0 to 2.9 for the left lens and from 12.7 to 2.9 Gy for the left lens for the same plan. Some automated plans provided significant decrease in lens doses; for example, plans #4, #8, #9, and #10 provided dose reductions of more than 3 Gy for both lenses, with a maximum reduction in plan #9 of 25.45 Gy for the left lens and 24.4 for the right lens. However, on a single occasion, Case #5, lens dose increased by 10.7 and 9.3 Gy for the left and right lens. Over all cases, the dose to the left eye was reduced by a mean of 3.55 Gy, by 1.32 Gy to the right eye and, for the lenses, the dose was reduced by a mean of 4.9 and 6.3 Gy.

**Figure 8:**
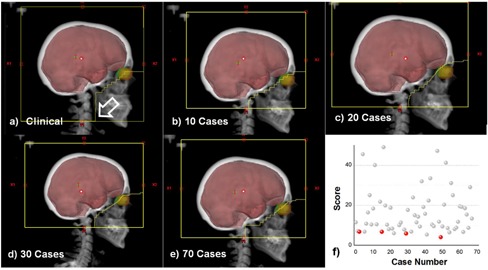
Solution geometry as function of database size. The clinical plan is shown in (a) and was judged as subclinical because the Y1 jaw is dropped too low (shown at arrow). The best automatched cases are shown for database sizes of 10 (b), 20(c), 30 (d), and 70 (e) cases. Corresponding scores for all database matches are shown in (f) as gray dots, with the best scores for the database configuration above marked in red.

Some cases were deemed suboptimal clinically because the collimator settings in the inferior directions was set too low or high in respect to the brain's inferior border. Such a scenario is presented in Fig. 8, with the incorrect collimator setting marked with an arrow. For this particular case, we show also dependence of the automated solution on the number of cases in the match database. In the same figure, the automated cases found for databases of size 10, 20, 30, and 70 cases. It should be noted that, for all automatched plans, the collimator setting is set as desired to a higher level when compared to the clinical plan.

Score dependence on the database size is detailed in Fig. 8(f), where scores for this test case are plotted as a function of database case number, with lower values indicating a better match. There are a many cases that are ranked at significantly higher scores and a few matches at similar lower scores. These solutions improve as the database size increases but do not differ significantly, suggesting that a smaller database may be used for whole brain treatments. For the case analyzed, the score after 10 cases was 6.82, after 20 cases was 6.79 at 30 cases was 5.93, while the final score when matching to 70 cases was 4.10. As a further improvement, we would like to create smarter database selection method where we cases are grouped by anatomical features so that the search does not check against similar database cases.

## IV. DISCUSSION

In the present report, we introduce an efficient method to segment the relevant anatomy for whole‐brain radiation therapy and then search a validated clinical database for prior cases with similar anatomy that could without modification be used for treatment. The process described resulted in an acceptable plan for the vast majority of cases without further intervention. The autoplans that were generated provided the necessary tradeoffs between target coverage and OAR sparing by using geometric and dosimetric information retrieved from a database of previous plans. Emphasis was placed on describing the search procedure and the associated metric to judge similarity between segmentations that match the patient's anatomy against a database of previously planned patients. To date, this approach was tested on clinical cases but not implemented in routine practice, with further work planned to incorporate the tool described in our institution's clinical routine and evaluate the usefulness of the automated segmentation and planning, as well as its practical benefits in reducing the workload and streamlining the planning process.

A fast and accurate segmentation is obtained by a combination of morphological filters in less than 1 min. The approach relies on isolating the soft tissue and then using morphological considerations to isolate the brain as the organ with the largest volume. The eyes and lenses are detected by similar consideration on the remaining soft tissue. This approach has the advantage that it is fast and simple as it does not require any extensive configuration, the CT dataset being the only datasets used as input by the segmentation procedure. After importing the plan in the treatment system, the user has only to specify the prescription dose that is not saved in the DICOM files and recompute the doses.

As a further development, we would like to extend the approach to other treatment sites and modalities. While the general concept would remain unchanged, the segmentation method has to be update to the organs of risk involved in the planning process, and the metric used to judge anatomical similarity should be customized to describe the specific criteria used to judge clinical plans. Furthermore, conformal treatment techniques, such as IMRT, would require larger expert case databases to capture the highly modulated beam and the significant changes in target shapes. While further research is needed to generalize the concept to other sites and techniques, this report, as well as previous investigations in prostate radiotherapy, suggests database mining is a valid and cost‐effective approach that will likely be employed in the future to simplify clinical practice.

## V. CONCLUSIONS

We presented a practical solution where a whole‐brain radiation therapy plan can be created automatically without any user interaction from simulation to evaluation. Segmentation of the CT dataset is performed using a combination of image morphology operators to delineate the eyes, lens, and brain and external contour. The segmentation is compared against a database of previously treated cases to find a case of similar anatomy. To increase speed and accuracy, the comparison is performed only on the eye and surrounding tissue that are critical in the planning process, ignoring the posterior brain segmentation where open fields are used. In 13 out of 15 cases, comparisons with the clinical plans show all autoplanned cases to provide the same target coverage for improvements in critical structures sparing.

## Supporting information

Supplementary Material
