# Supplementary material for: Automated population‐based planning for whole brain radiation therapy
Source: J Appl Clin Med Phys. 2015 Sep 8;16(5):76–86. doi: 10.1120/jacmp.v16i5.5258 (PMC5690177; doi:10.1120/jacmp.v16i5.5258)
Supplement: Supplementary file 1 — Supplementary Material [file ACM2-16-076-s001.docx]

**Automated Population-Based Planning for Whole Brain Radiation Therapy**

E. Schreibmann Ph.D.^a)^, T. Fox Ph.D.

W. Curran M.D., H. K. Shu M.D. Ph.D., I. Crocker M.D.,

Department of Radiation Oncology, Emory University School of Medicine,

Atlanta, Georgia 30306, USA

a) Author to whom correspondence should be addressed.

Emory University School of Medicine

Department of Radiation Oncology

1365 Clifton Road NE

Atlanta, GA 30306

E-mail: anees.dhabban@radonc.emory.org

Phone: (404) 778 5667

Fax: (404) 778 4139

Submitted to: *Journal of Applied Clinical Medical Physics*
